# The Impact of Physical Effort on the Gut Microbiota of Long-Distance Fliers

**DOI:** 10.3390/microorganisms11071766

**Published:** 2023-07-06

**Authors:** Elís Domingos Ferrari, Bruno César Miranda Oliveira, Hannah N. Creasey, Débora R. Romualdo da Silva, Alex Akira Nakamura, Katia D. Saraiva Bresciani, Giovanni Widmer

**Affiliations:** 1 União das Facultades dos Grandes Lagos (UNILAGO), São José do Rio Preto 15030-070, São Paulo, Brazil; elisd.ferrari@yahoo.com.br (E.D.F.); bruno.9988@hotmail.com (B.C.M.O.); 2 Cummings School of Veterinary Medicine, Tufts University, North Grafton, MA 01536, USA; hannah.creasey@tufts.edu (H.N.C.); debora.silva@tufts.edu (D.R.R.d.S.); 3 School of Veterinary Medicine, São Paulo State University (UNESP), Araçatuba 16050-680, São Paulo, Brazil; alex.nakamura@unesp.br (A.A.N.); katia.bresciani@unesp.br (K.D.S.B.)

**Keywords:** *Columbia livia*, 16S amplicon sequencing, principal coordinate analysis, metabolic pathways, *mothur*, PICRUSt2

## Abstract

Flying pigeons (*Columbia livia*) are extensively studied for their physical endurance and superior sense of orientation. The extreme physical endurance of which these birds are capable creates a unique opportunity to investigate the possible impact of long-distance flying on the taxonomy and metabolic function of the gut microbiota. This project was enabled by access to two groups of pigeons raised by the same breeder in the same conditions, except that one group was trained in long-distance flying and participated in multiple races covering a total distance of over 2600 km over a 9-week period. In contrast, the second group did not fly. The fecal microbiota was analyzed using 16S amplicon sequencing, and the taxonomy and metabolic function were inferred from this sequence data. Based on phylogenetic distance and metabolic function, flying and non-flying pigeons were found to harbor distinct bacterial microbiota. The microbiota taxonomy varied extensively between the birds, whereas the inferred metabolic potential was relatively stable. Age was not a significant determinant of the fecal microbiota profile. In flying birds, the metabolic pathways annotated with biosynthesis were enriched, representing 60% of the 20 metabolic pathways that were most closely associated with flying.

## 1. Introduction

Flying pigeons, also known as homing pigeons, have long attracted the interest of biologists for their sense of orientation, homing ability and physical endurance [1,2]. Pigeons are known for their orientation skills and their instinct to return to the pigeonry where they were born. Pigeon breeding and racing are popular activities in certain parts of the world. The state of Minas Gerais in Central Brazil is home to many pigeon breeders who compete with their birds in long-distance races. To minimize casualties, the races take place each year during the dry season between May and September. Competitions over less than 500 km take place weekly, whereas longer races are separated by a 2-week period. The winner of the races is determined based on the sum of the scores obtained by each breeder’s birds over multiple races of increasing distance. The breeders generally prefer racing unmated females because they believe these birds have a superior flight performance compared to males. This belief is supported by a study that measured speed and flight efficiency, defined as the linear distance between the release point and home divided by the actual distance traveled by the pigeons between these two points [3]. In this study, unmated females flying solo averaged a speed of 71 km/h, faster than solo flying males or mated females.

The recent explosion in the range and scope of research on host-associated bacterial populations or “microbiota” has uncovered different mechanisms of interaction between microbiota and the host, highlighting the importance of bacterial metabolism to the immune system [4], resistance to enteric infections [5], behavior [6] and other traits. Compared to numerous analyses of chicken microbiota, published studies focused on the microbiota of other domestic or wild avian species are relatively rare [7,8,9,10].

Motivated by the interest in understanding the effect of exercise on the intestinal microbiota and the possibility of improving athletic performance with microbiota-centered nutrition [11], many studies have compared the fecal microbiota of athletes and sedentary individuals, athletes and non-competing controls [12,13] or even athletes competing in different disciplines [14]. Studies with human volunteers have tested the effect of one or multiple sessions of intense exercise on the gut microbiota. For instance, a prospective study on the effect of short-term exercise on the fecal microbiota found no detectable impact except for a change in the composition of the virome [15]. Similarly, an intense treadmill exercise with trained athletes found no difference in microbiota profiles when comparing samples collected before and within a few hours of exercising. Examining the long-term impact of physical activity on the intestinal microbiota, O’Donovan et al. observed differences in the functional repertoire of the fecal microbiota of athletes competing in different disciplines [14]. A cohort of cross-country skiers participating in continuous training and competition was studied by Hintikka et al. [16]. This study found no difference in microbiota α diversity between athletes and controls. A similar line of research involves the administration of probiotics or other microbiota-targeting food and measuring physical performance in individuals receiving actual supplements vs. placebo controls [17]. Regardless of the design of the trials, the inability to control the diet and lifestyle of human subjects limits the statistical power of the intervention and raises the possibility that useful information could be gained from animal models.

Here, we describe a study of two cohorts of flying pigeons: one participating in periodic training and long-distance races and the other consisting of non-flying birds housed in the same facility. We report on the analysis of the taxonomy and metabolic function of the fecal microbiota of these birds and identify the taxonomic and functional differences associated with flying status.

## 2. Materials and Methods

### 2.1. Birds

The racing pigeons (*Columbia livia*) belonged to a single pigeonry of 190 birds, which is part of the Pigeon Breeder Association of Formiga, located in Formiga, Minas Gerais State, Brazil (20.4606° S, 45.4281° W). Only 30 birds of this flock were studied. The pigeons were fed ad libidum a grain mixture sold under the name of Mistura Premium by Moura (Contagem, Minas Gerais, Brazil). The grain mixture contained 140 g/kg protein, 85 g/kg fiber, 35 g/kg minerals, 0.65–1.1 g/kg calcium and 3.7 g/kg phosphorous. The sampled pigeons belonged to two groups housed separately in the same pigeonry (Figure S1); a total of 15 birds raised for competing and the same number raised for reproduction were included. Typically, reproductive birds are retired fliers and may have been purchased from another flock. In 2019, the year the pigeons were sampled for this study, the pigeons in the flying cohort participated in 3 training races followed by 8 competitions. The cumulative straight-line distance between the point of release and Formiga flown by each pigeon between May and July 2019 was 2669 km. The breeder participating in this study decided to stop racing before the end of the 2019 season, and the last flight his racing pigeons participated in was on 21 July over a distance of 436 km. In addition to the training flights and races, the flying cohort underwent daily flights averaging 60 min (Supplementary Video S1). Table 1 summarizes the flight schedule. The non-flying birds did not participate in any training flights or competitions.

Two fecal samples were collected from each bird: the first on 28 July 2019 and the second one week later on 4 August 2019. Feces were collected from female birds only. The pigeons were placed individually in a cardboard box for a few minutes until they defecated. After defecating, the birds were placed back in their usual housing together with the flock. One of the reproductive pigeons escaped between the first and second collection, and therefore, only one sample was available from this bird. Feces were collected from the bottom of the box with a disposable wooden spatula. The samples were transferred to 2 mL microcentrifuge tubes and stored at −20 °C without delay. The fecal samples were extracted using the GenElute™ Stool DNA Isolation kit (Sigma-Aldrich, St. Louis, MO, USA) according to the manufacturer’s protocol, except that a single 10 min homogenization step was applied instead of the recommended 3 min. The extracted DNA was stored at −20 °C.

### 2.2. Molecular Biology

The dual barcode protocol described by Kozich et al. [14] was used to amplify the V4 region of the 16S rRNA gene and uniquely barcode each amplicon. The amplicon concentration was estimated using a Qubit 3 fluorometer (ThermoFischer Scientific, Waltham, Massachusetts). The amplicons were combined at approximately equal concentration. The library was size-selected with the Pippin Prep system. The library was sequenced paired-end 500 cycles on a MiSeq instrument operated by the Tufts Genomics core (tucf.org, accessed 4 July 2023).

### 2.3. Bioinformatics

Aamplicons that generated fewer than 5000 read pairs were excluded. Amplicons with more than 100,000 read pairs were subsampled to 100,000 using the program sub.sample in *mothur* [15]. No other normalization was applied. A total of 46 V4 amplicons were included in the bioinformatics analysis. The amplicons averaged 66,900 read pairs (range = 5148–100,000, mean = 66,900, standard deviation (SD) = 33,335) (Table S1). Sequence de-noising was performed in *mothur* essentially as described [16,17]. Operational Taxonomic Units (OTUs) were formed with a 3% sequence dissimilarity cut-off based on the OptiClust method [18]. OTUs with an average of less than 1 sequence per sample were removed. Sample clustering was tested using ANOSIM [19] as implemented in *mothur*. Bonferroni correction was applied for multiple comparisons. Sequences were classified against the Silva reference taxonomy [20] (release 138.1) using classify.seqs. For most analyses, a 75% cut-off threshold was applied. To identify the bacterial taxa that differed significantly between the experimental groups, LDA [21] was performed using the program LefSe [22] as implemented in *mothur*. Canonical correspondence analysis (CCA) and redundancy analysis (RDA) were used to assess the impact of one or multiple independent variables (predictors) on the microbiota. The choice of constrained ordination method depended on whether the dependent variables were best modelled by a linear or a unimodal model [23]. The pseudo-F statistic [24] was calculated to test the null hypothesis of no association between the dependent and independent variables. CCA and RDA were performed in CANOCO, release 5.15 [23]. Shannon diversity indices were calculated using the program summary.single in *mothur*. Metabolic pathways abundance was inferred from the 16S sequence data using PICRUST2 [25]. The PICRUST2 pipeline requires a biom-formatted input file specifying the abundance of each pathway in each sample. This file was created as follows. A “shared” data matrix was generated in *mothur* from a fasta file using the make.shared command with argument label = ASV. This file was converted into a biom file using biom convert [26]. Pathways with ≤1 occurrence per sample were culled. The distance between each pair of samples was calculated in GenAlEx [27] from the pathway abundance values using the “haploid SSR” distance metric. This distance is equal to the squared difference between the abundance values for each pathway summed over all pathways.

### 2.4. Quality Controls

To assess the quality of the extraction, PCR and sequencing procedures, one pigeon sample (#13) was duplicated by tagging each of two amplicons obtained from this sample with a unique pair of barcodes. The β diversity between the sequences obtained from these samples, measured using the weighted UniFrac metric [28], was 0.054. Further, 5 amplicons were generated in a separate PCR from DNA extracted from a synthetic bacterial population (BEI Resources, Manassas, VA, cat no. HM-782D). The ten weighted UniFrac distances between these 5 samples were 0.033, 0.036, 0.018, 0.036, 0.027, 0.035, 0.031, 0.028, 0.030 and 0.025. The mean of these distance values equals 0.03 and represents 4.9% of the average. The UniFrac distance was 0.61 between the 46 bird samples analyzed here (1035 pairwise distance values). The mean phylum-level proportion for these five control samples was 0.041 (SD = 0.001) for Deinococcus-Thermus, 0.0433246 (SD = 0.002) for Actinobacteria, 0.37 (SD = 0.006) for Proteobacteria, 0.488 (SD = 0.008) for Firmicutes, 0.048 (SD = 0.003) for Bacteroidetes and 0.005 (SD = 0.002) for unclassified bacteria. The expected proportions for the same 6 phyla were 0.05, 0.10, 0.30, 0.50, 0.05 and 0, respectively.

## 3. Results

### 3.1. Global Analysis of 16S Sequences and Inferred Metabolic Pathways

**Figure 1:**
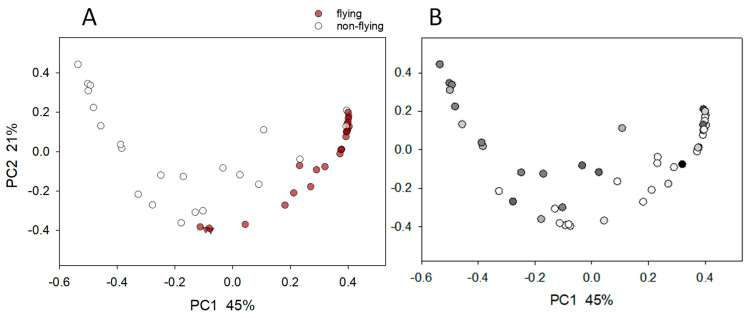
Principal coordinate analysis of 46 pigeon fecal microbiota. (**A**) Datapoints colored according to exercise status. Triangles at PC1 = −0.076/PC2 = −0.396 and PC1 = −0.093/PC2 = −0.396, respectively, represent duplicated samples of pigeon #13, as described in the Materials and Methods section. The distance between these datapoints is 0.054 weighted UniFrac units and is a measure of technical variation. (**B**) Same PCoA as shown in panel A, but datapoints are shaded to represent age. White symbols represent 2 years of age, and black represents 12 years. Note segregation by flying status but not by age.

**Figure 2:**
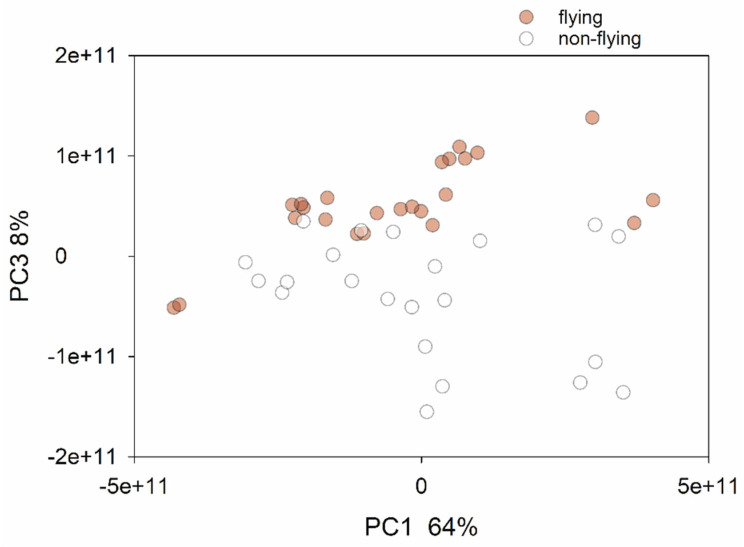
Principal coordinate analysis of pairwise distances based on metabolic pathway abundance values. A better visual separation of the two groups is obtained in the PC1–PC3 plane than in the PC1–PC2 plane.

Unconstrained ordination analysis was applied to visualize the pairwise β diversity between the samples based on the weighted UniFrac distances (Figure 1). This analysis revealed a clear effect of flying on the profile of the pigeons’ microbiota. The clustering of samples according to flying status was statistically significant (ANOSIM, R = 0.25, *p* < 0.0001). The analogous analysis of the metabolic pathway abundance from the same 46 samples also revealed a clear segregation according to the flying status (Figure 2), implying that, in these birds, physical exercise selected for a distinct intestinal microbiota with respect to taxonomy and metabolic function. The flying birds were significantly younger (mean = 3.2 years, SD = 2.54) than those that did not fly (mean = 6.07, SD = 2.68, Rank Sum test *p* = 0.005). To confirm the conclusion that the fecal microbiota profiles segregate according to flying status, it was important to test whether the flying status remained a significant variable after subtracting the effect of age. For this analysis, we used CCA with flying status as independent variables and age as a covariate. Given the large variation in sequence depth between the amplicons described in the Material and Methods section, the sequence count was also included as a second covariate. Consistent with the microbiota profile being impacted by flying status, CCA returned a significant pseudo-F value of 4.7 (*p* = 0.0001). This outcome confirms the association of flying status with the fecal microbiota profile and shows that this effect is independent of age and sequencing depth. Consistent with this interpretation, CCA with age as an independent variable returned a non-significant effect on the sequence profile (pseudo-F = 1.4, *p* = 0.102).

**Figure 3:**
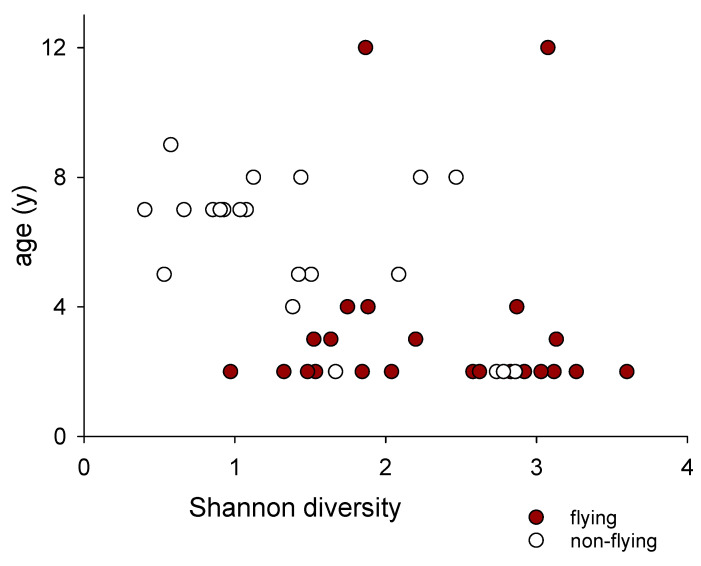
Shannon diversity in flying and non-flying birds according to age. α diversity is based on the relative abundance of 826 OTUs meeting the threshold of ≥1 per sample or ≥46 in the entire dataset. The correlation between age and diversity is statistically significant (Pearson r = −0.43, *n* = 46, *p* = 0.003). Note the high diversity of the two samples originating from a 12-year-old flying pigeon.

We compared the OTU α diversity between the flying birds and non-flying birds. Based on the Shannon and Berger–Parker diversity metrics, the microbiota of the flying birds was, on average, more diverse than the microbiota of their non-flying flock mates. The Shannon diversity in the former group averaged 2.3 (*n* = 24, SD = 0.73). In the latter group, a mean diversity of 1.4 (*n* = 22, 0.77) was obtained (*t*-test, *p* = 0.0002). The results based on the Berger–Parker index similarly showed that the microbiota from the flying pigeons was more diverse (Mann–Whitney Rank Sum test *p* = 0.017). A significant negative correlation between α diversity and age was observed (Figure 3). Of potential interest is the finding that the α diversity of the microbiota from a 12-year-old flying bird (#8) was relatively high and comparable to the diversity of a younger flying bird. This observation suggests that exercise promotes bacterial diversity regardless of a bird’s age, but this conclusion remains tentative as it is based on a single bird.

### 3.2. Metabolic Pathways Enrichment Analysis

The functional repertoire of the microbiota in the flying and non-flying pigeons was inferred from the 16S sequence data using PICRUSt2 [18]. LDA was applied to identify the 20 metabolic pathways with the largest LDA score in the flying and non-flying groups. The number of pathways included in this analysis, i.e., 40 with the highest LDA score, was chosen arbitrarily out of a total of 126 pathways, which are significantly associated with flying/not flying. For these 40 pathways, the annotations were downloaded from MetaCyc [19] together with the annotation of the 1022 bacterial metabolic pathways found in this database. Table 2 shows the proportion of the three common metabolic functions (biosynthesis, degradation, fermentation) in the flying and non-flying groups and in the 1022 MetaCyc pathways.

The comparison of the flying and non-flying pathway data with the entire MetaCyc’s 1022 bacterial pathway collection shows a different metabolic repertoire in the flying and non-flying birds. In the flying pigeons, the pathways related to biosynthesis were particularly common; a total of 60% (12/20) of the pathways were annotated with this term as compared to 30% in the non-flying birds and 38% in the entire MetaCyc collection. The proportion of flying and non-flying pathways differed significantly from the MetaCyc database (*p* < 0.005 and <0.05, respectively). The flying and non-flying categories were also significantly different from each other (Chi-Square = 9.955, 3 d.f., *p* = 0.019).

### 3.3. Temporal Evolution between Repeated Collections of Feces

**Figure 4:**
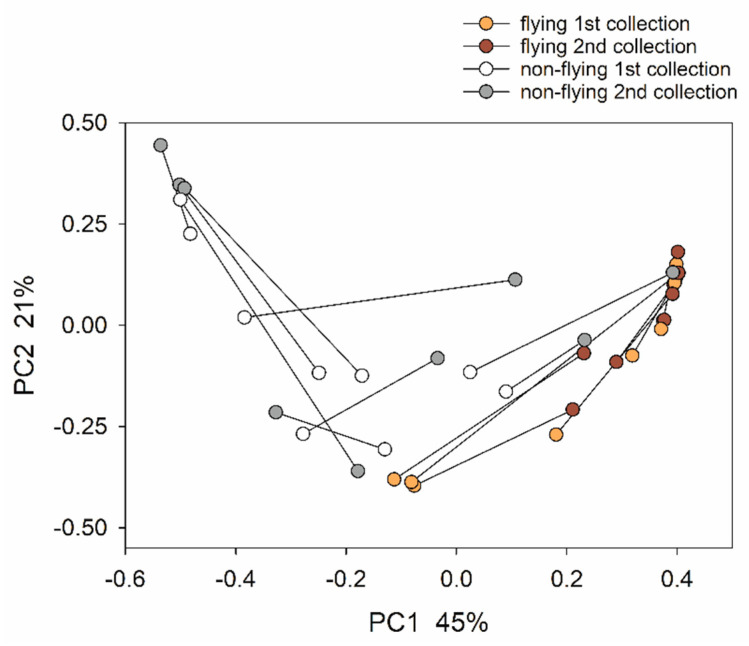
Principal coordinate analysis visualizing the trajectory of the pigeon microbiota between the 1st and 2nd collection. The PCoA is based on the weighted UniFrac distances and represents changes in microbiota taxonomy. Datapoints from the same bird are connected. Colored datapoints are from flying birds, and white/shaded points are from non-flying pigeons. White/orange indicates first collection; grey/dark red indicates 2nd collection. The distance separating datapoints from the same bird is a measure of microbiota change in the time between feces collection. Variation represented by each axis is indicated as percent of total variation.

**Figure 5:**
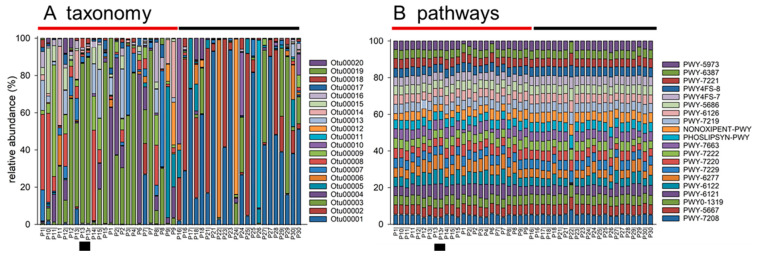
Taxonomically variable microbiota encodes similar metabolic functions. Each stack represents one sample. The 20 most abundant OTUs (**A**) and metabolic pathways (**B**) are represented with different colors. Pipe symbol (|) in the sample names shown below the plots indicates second collection. Topmost horizontal bars indicate samples from pigeons that fly (brown) and pigeons that do not (black). Small black rectangle below each plot indicates pigeon #13 replicated samples. The consensus taxonomy of the OTUs and the name of each metabolic pathway are shown in Table S2.

The collection of two samples from each bird one week apart enabled a comparison of intra-bird vs. inter-bird distances, both at the level of microbiota taxonomy and inferred metabolic function. Particularly for the birds that did not fly, the weighted UniFrac distances between the samples collected from the same pigeon were surprisingly large, averaging 0.66 (nine comparisons, SD = 0.15). The microbiota of the flying birds was more stable (Figure 4) (mean UniFrac distance = 0.36, SD = 0.22), which represents a significant difference between the flying and non-flying groups with respect to microbiota stability (*t*-test, *n* = 9 per group, *p* = 0.004). To assess the functional significance of the temporal changes in the microbiota populating the same bird, a corresponding analysis of pairwise distances was performed based on the inferred metabolic pathway abundance data. This analysis revealed that, contrary to expectations, on a functional level, samples from the same pigeon were not more similar to each other than samples from different pigeons (Mann–Whitney Rank Sum test, *p* = 0.74). When comparing the intra-pigeon pairwise distances in contrast to the UniFrac-based analysis, the change in the pathway profile in the flying birds was similar to the change in the non-flying birds (*t*-test, *p* = 0.80). Figure 5 illustrates the contrast between the taxonomic and functional stability for the 20 most abundant taxa and pathways. Taken together, these observations indicate that temporal taxonomic changes do not translate into functional changes, and the metabolic repertoire of a bird’s microbiota is relatively stable regardless of physical activity.

### 3.4. No Impact of Uncontrolled Variables

Unequal amplification efficiency, as is the case with the samples analyzed here, is commonly observed, particularly with samples collected in the field. This variability was reflected in a relatively wide range of sequence yield per amplicon, summarized in the Materials and Methods section and in Table S1. It was, therefore, important to exclude the possibility that sequence depth, i.e., the number of reads per amplicon, impacted the outcome of the analyses. First, we examined whether the sequence yield was different between the samples collected from the flying and non-flying birds. This analysis returned a negative result (Mann–Whitney Rank Sum Test, *p* = 0.17; *n* = 24 flying, *n* = 22 non-flying). Second, sequence yield was incorporated as a covariate in the CCAs focused on the effect of flying on microbiota, OTU abundance and on the inferred metabolic pathway abundance. By defining the sequence yield as a covariate, the effect of this variable was subtracted. The association between flying activity and OTU or pathway abundance described above (Section 3.1) remained significant, regardless of whether the sequence yield was included as a covariate or not. This result confirms that the sequence yield did not impact the results. Third, consistent with these outcomes, CCA using sequence yield as the sole explanatory variable showed no significant association of this variable with the microbiota profile (pseudo-F = 1.5, *p* = 0.07).

As apparent in Figure 5A and Table S2, a large proportion of sequences clustered in Otu00001 remained unclassified using a 75% cut-off. Considering the absence of OTUs classified as Firmicutes at this confidence level, we examined whether the 9765 unique unclassified sequences represented PCR artifacts, like PCR chimeras, or sequences that are not found in the reference taxonomy used for classification [20]. The search for chimeras [21] among the unclassified sequences flagged 1040 unique sequences, equivalent to 1.1% of the total number of unique sequences (100% = 95,898) included in the entire dataset or equivalent to the 10.6% of the unclassified sequences. The taxonomic classification of the 9765 sequences using a lower cut-off of 60%, instead of 75%, assigned 95.7% of these sequences to the phylum Firmicutes. However, almost the totality of these reads remained unclassified at the class level, suggesting the presence in the pigeons of bacteria that are absent from the Silva reference taxonomy.

## 4. Discussion

### 4.1. Motivation

Because of the importance of the intestinal microbiota for extracting energy from food, the potential link between microbial metabolism and physical endurance is being investigated. This research is conducted primarily in human athletes and in laboratory rodents. The production of short-chain fatty acids resulting from the fermentative metabolism of dietary fiber in anaerobic bacteria has been highlighted as a possible mechanism by which the microbiota in the gut may impact endurance [22]. These molecules are important as a source of energy for intestinal enterocytes leading to the hypothesis that SCFA may play a role in linking microbiota metabolism and physical endurance. A clear picture of if and how the intestinal microbiota promotes or diminishes physical endurance has not yet emerged.

A primary motivation of this study is the unique features of homing pigeons, primarily the extreme flight performance and the ease with which these birds can be handled. Migrating birds are comparable with respect to extreme physical endurance, but logistical difficulties for sampling, tracking and recapturing limit the research on migratory birds and their intestinal microbiota [23,24]. The fact that the diet of pigeons raised for flying and racing is carefully controlled represents an additional advantage over wild birds.

### 4.2. Unique Features of the Pigeon Model

The evolutionary distance between birds and mammals limits the use of birds to model human physiology. A clear advantage of the experimental setting described here is that flying and non-flying birds were housed in the same facility and were fed the same grain mixture. This setting amounts to a nearly ideal experimental setup, which eliminated many uncontrolled variables, enabling us to test for statistically significant associations between sustained and intense exercise and the microbiota. Experiments with rodents have investigated similar questions related to microbiota and endurance [25,26]. The level of exercise in these studies is, however, limited by ethical considerations, as forced physical activity beyond a short duration may not be acceptable. Such experiments are also likely to be impacted by the stress experienced by animals forced to sustain physical activity and the potential for stress to impact the gut microbiota.

We tentatively interpret the differences between the microbiota of flying and non-flying pigeons described here as resulting from repeated bouts of intense exercise as opposed to the microbiota influencing flight performance. This interpretation is based on the fact that flying pigeons are not assigned to the flying cohort strictly based on previous performance. Instead, breeders consider appearance together with flight performance to select birds for reproduction and purchase birds for reproduction from other breeders. Competing birds also may “retire” to the reproductive flock as they age. This complex breeding design is not expected to exert a selective pressure for enhanced physical endurance on the microbiota. To establish causality more directly, specific changes expected to impact physical endurance would have to be induced in the birds’ microbiota, and the impact of such treatments on flight performance would have to be evaluated.

### 4.3. Taxonomy

The observed difference in taxonomic vs. functional variability in the pigeons’ microbiota was to some extent expected based on similar observations in humans [27]. More surprising is the taxonomic diversity between the birds’ microbiota, given the constant diet and cohabitation within the flying and non-flying flocks. The large diversity between the two samples collected from the same bird, particularly from non-flying pigeons, was also unexpected but may be explained by the relatively low microbiota α diversity. Indeed, a positive correlation between low α diversity and instability has been reported from a study on human subjects [28]. Shannon diversity values in the 2–3 range found in our samples would be considered abnormally low in humans and in laboratory rodents, perhaps explaining the extensive intra-bird β diversity. The presence among the flying birds of a 12-year-old pigeon revealed a possible trend for microbiota diversity to be associated with physical activity rather than age. In fact, pigeon 8 stood out among the flying flock for its age. The Shannon and Berger–Parker diversities measured for this bird were within the range of flying pigeons and higher than pigeons of similar age.

The observed high abundance of Proteobacteria in the microbiota of certain pigeons is noteworthy but not unprecedented. Boukerb et al. similarly found microbiota rich in Proteobacteria in waterfowl and in chickens [29]. Similarly, high relative abundances of Proteobacteria were observed in migratory warblers [23] and in migratory geese [30]. The detection of a large proportion of sequences classified as Firmicutes, albeit with lower statistical confidence, suggests the presence in pigeons of Firmicutes taxa that are not represented in the Silva reference taxonomy [31]. Given the importance of Firmicutes for the digestion of dietary fiber, these bacteria may be worth further identification using metagenomic methods with higher taxonomic resolution.

## 5. Conclusions

The analysis of the fecal microbiota of flying and non-flying pigeons belonging to the same breeder has revealed taxonomical and functional differences associated with flying and racing. The clustering of microbiota into these two groups was reflected in a higher α diversity and temporal stability in flying birds. The metabolic pathways encoded by the microbiome of flying birds were enriched in biosynthetic functions. The results raise interesting questions related to the mechanistic link between the intestinal microbiota and the remarkable physical effort of which homing pigeons are capable. A better understanding of the link between gut and physical endurance will require moving from relative taxonomic abundances obtained with 16S amplicon sequencing to absolute abundances. Applying metagenomic tools with a higher taxonomic resolution will also advance our understanding of the link between the microbiota’s metabolic functions and physical activity.

## Figures and Tables

**Table 1:** 2019 training and racing schedule.

Race Modality	Site of Release ^1^	Longitude	Latitude	Straight-Line Distance to Destination (km)	Date
Training	Lagoa da Prata	45.5353° W	20.0272° S	53	12 May 19
Training	Luz	45.6815° W	19.7927° S	75	19 May 19
Training	Bom Despacho	45.3108° W	19.5779° S	98	26 May 19
Competition	Pompéu	45.0057° W	19.2237° S	144	2 June 19
Competition	Curvelo	44.4307° W	18.7532° S	216	9 June 19
Competition	Corinto	44.4521° W	18.3647° S	249	16 June 19
Competition	Augusto Lima	44.2100° W	18.0719° S	294	23 June 19
Competition	Joaquim Felício	44.1707° W	17.7589° S	324	30 June 19
Competition	Engenheiro Navarro	43.9515° W	17.2873° S	378	7 July 19
Competition	Bocaiúva	43.8203° W	17.1113° S	402	14 July 19
Competition	Montes Claros	43.8582° W	16.7286° S	436	21 July 19

^1^ State of Minas Gerais.

**Table 2:** Pathway enrichment analysis based on the observed and expected proportions of pathway annotation terms ^1^.

	MetaCyc	Flying	Non-Flying
biosynthesis	0.383	0.600	0.300
degradation	0.443	0.150	0.250
fermentation	0.015	0.150	2 × 10^−6^
none of above	0.159	0.10	0.450
total	1	1	1
G test ^2^ statistic (*p*)		16.4 (*p* < 0.005)	10.2 (*p* < 0.05)

^1^ 20 pathways with highest LDA score in flying and non-flying samples (40 total). ^2^ G Test for Goodness-of-Fit using MetaCyc database as the reference.

## Data Availability

Sequences were deposited in FASTQ format in the Sequence Read Archive, National Library of Medicine, National Center for Biotechnology Information, under project accession number PRJNA967748.

## Supplementary Materials

The following supporting information can be downloaded at: https://www.mdpi.com/article/10.3390/microorganisms11071766/s1, Figure S1: Inside the pigeonry; Table S1: Samples, age, fly status and sequence yield; Table S2: Taxonomy of OTU 1–20 and metabolic pathways 1–20 represented in Figure 5; Video S1: Pigeons’ daily training.

## References

[1] Mehlhorn J., Rehkämper G. (2011). Brieftauben: Navigationskünstler und Spitzensportler. Biol. Unserer Zeit.

[2] Michener M.C., Walcott C. (1967). Homing of single pigeons—Analysis of tracks. J. Exp. Biol..

[3] Mehlhorn J., Rehkaemper G. (2016). The Influence of Social Parameters on the Homing Behavior of Pigeons. PLoS ONE.

[4] Dang A.T., Marsland B.J. (2019). Microbes, metabolites, and the gut–lung axis. Mucosal Immunol..

[5] Desai M.S., Seekatz A.M., Koropatkin N.M., Kamada N., Hickey C.A., Wolter M., Pudlo N.A., Kitamoto S., Terrapon N., Muller A. (2016). A Dietary Fiber-Deprived Gut Microbiota Degrades the Colonic Mucus Barrier and Enhances Pathogen Susceptibility. Cell.

[6] Vuong H.E., Yano J.M., Fung T.C., Hsiao E.Y. (2017). The microbiome and host behavior. Annu. Rev. Neurosci..

[7] Sun F., Chen J., Liu K., Tang M., Yang Y. (2022). The avian gut microbiota: Diversity, influencing factors, and future directions. Front. Microbiol..

[8] Herder E.A., Skeen H.R., Lutz H.L., Hird S.M. (2023). Body Size Poorly Predicts Host-Associated Microbial Diversity in Wild Birds. Microbiol. Spectr..

[9] Lu Z., Li S., Wang M., Wang C., Meng D., Liu J. (2022). Comparative Analysis of the Gut Microbiota of Three Sympatric Terrestrial Wild Bird Species Overwintering in Farmland Habitats. Front. Microbiol..

[10] Oliveira B.C., Murray M., Tseng F., Widmer G. (2020). The fecal microbiota of wild and captive raptors. Anim. Microbiome.

[11] Hughes R.L., Holscher H.D. (2021). Fueling Gut Microbes: A Review of the Interaction between Diet, Exercise, and the Gut Microbiota in Athletes. Adv. Nutr..

[12] Barton W., Penney N.C., Cronin O., Garcia-Perez I., Molloy M.G., Holmes E., Shanahan F., Cotter P.D., O’Sullivan O. (2018). The microbiome of professional athletes differs from that of more sedentary subjects in composition and particularly at the functional metabolic level. Gut.

[13] Wiacek J., Szurkowska J., Krysciak J., Galecka M., Karolkiewicz J. (2023). No changes in the abundance of selected fecal bacteria during increased carbohydrates consumption period associated with the racing season in amateur road cyclists. PeerJ.

[14] O’Donovan C.M., Madigan S.M., Garcia-Perez I., Rankin A., O’ Sullivan O., Cotter P.D. (2020). Distinct microbiome composition and metabolome exists across subgroups of elite Irish athletes. J. Sci. Med. Sport.

[15] Cronin O., Barton W., Skuse P., Penney N.C., Garcia-Perez I., Murphy E.F., Woods T., Nugent H., Fanning A., Melgar S. (2018). A Prospective Metagenomic and Metabolomic Analysis of the Impact of Exercise and/or Whey Protein Supplementation on the Gut Microbiome of Sedentary Adults. mSystems.

[16] Hintikka J.E., Munukka E., Valtonen M., Luoto R., Ihalainen J.K., Kallonen T., Waris M., Heinonen O.J., Ruuskanen O., Pekkala S. (2022). Gut Microbiota and Serum Metabolome in Elite Cross-Country Skiers: A Controlled Study. Metabolites.

[17] Smarkusz-Zarzecka J., Ostrowska L., Leszczyńska J., Orywal K., Cwalina U., Pogodziński D. (2020). Analysis of the Impact of a Multi-Strain Probiotic on Body Composition and Cardiorespiratory Fitness in Long-Distance Runners. Nutrients.

[18] Douglas G.M., Maffei V.J., Zaneveld J.R., Yurgel S.N., Brown J.R., Taylor C.M., Huttenhower C., Langille M.G.I. (2020). PICRUSt2 for prediction of metagenome functions. Nat. Biotechnol..

[19] Caspi R., Billington R., Keseler I.M., Kothari A., Krummenacker M., Midford P.E., Ong W.K., Paley S., Subhraveti P., Karp P.D. (2020). The MetaCyc database of metabolic pathways and enzymes—A 2019 update. Nucleic Acids Res..

[20] Quast C., Pruesse E., Yilmaz P., Gerken J., Schweer T., Yarza P., Peplies J., Glockner F.O. (2013). The SILVA ribosomal RNA gene database project: Improved data processing and web-based tools. Nucleic Acids Res..

[21] Rognes T., Flouri T., Nichols B., Quince C., Mahe F. (2016). VSEARCH: A versatile open source tool for metagenomics. PeerJ.

[22] Bongiovanni T., Yin M.O.L., Heaney L.M. (2021). The athlete and gut microbiome: Short-chain fatty acids as potential ergogenic aids for exercise and training. Int. J. Sports Med..

[23] Skeen H.R., Cooper N.W., Hackett S.J., Bates J.M., Marra P.P. (2021). Repeated sampling of individuals reveals impact of tropical and temperate habitats on microbiota of a migratory bird. Mol. Ecol..

[24] Kreisinger J., Kropackova L., Petrzelkova A., Adamkova M., Tomasek O., Martin J.F., Michalkova R., Albrecht T. (2017). Temporal Stability and the Effect of Transgenerational Transfer on Fecal Microbiota Structure in a Long Distance Migratory Bird. Front. Microbiol..

[25] Lamoureux E.V., Grandy S.A., Langille M.G. (2017). Moderate exercise has limited but distinguishable effects on the mouse microbiome. mSystems.

[26] Yun E.-J., Imdad S., Jang J., Park J., So B., Kim J.-H., Kang C. (2022). Diet Is a Stronger Covariate than Exercise in Determining Gut Microbial Richness and Diversity. Nutrients.

[27] Koren O., Goodrich J.K., Cullender T.C., Spor A., Laitinen K., Backhed H.K., Gonzalez A., Werner J.J., Angenent L.T., Knight R. (2012). Host remodeling of the gut microbiome and metabolic changes during pregnancy. Cell.

[28] Jeffery I.B., Lynch D.B., O’Toole P.W. (2016). Composition and temporal stability of the gut microbiota in older persons. ISME J..

[29] Boukerb A.M., Noel C., Quenot E., Cadiou B., Cheve J., Quintric L., Cormier A., Dantan L., Gourmelon M. (2021). Comparative Analysis of Fecal Microbiomes From Wild Waterbirds to Poultry, Cattle, Pigs, and Wastewater Treatment Plants for a Microbial Source Tracking Approach. Front. Microbiol..

[30] Cho H., Lee W.Y. (2020). Interspecific comparison of the fecal microbiota structure in three Arctic migratory bird species. Ecol. Evol..

[31] Glöckner F.O., Yilmaz P., Quast C., Gerken J., Beccati A., Ciuprina A., Bruns G., Yarza P., Peplies J., Westram R. (2017). 25 years of serving the community with ribosomal RNA gene reference databases and tools. J. Biotechnol..

