# Supplementary material for: The Impact of Physical Effort on the Gut Microbiota of Long-Distance Fliers
Source: Microorganisms. 2023 Jul 6;11(7):1766. doi: 10.3390/microorganisms11071766 (PMC10386721; doi:10.3390/microorganisms11071766)
Supplement: Supplementary file 1 [file microorganisms-11-01766-s001.zip › TableS2.pdf]

Taxonomy of OTU 1-20 represented in Fig. 5. Following the OTU number, the sequence count and the complete classification is shown. The numbers in parentheses indicates the classification bootstrap value.

Otu00001, 591467, Bacteria_unclassified(100);Bacteria_unclassified(100);Bacteria_unclassified(100);Bacteria_unclassified(100);Bacteria_unclassified(100);Bacteria_unclassified(100);Bacteria_unclassified(100);Bacteria_unclassified(100);

Otu00002, 355439, Proteobacteria(100);Gammaproteobacteria(100);Cardiobacteriales(100);Cardiobacteriaceae(100);Cardiobacteriaceae_unclassified(99);Cardiobacteriaceae_unclassified(99);Cardiobacteriaceae_unclassified(99);Cardiobacteriaceae_unclassified(99);

Otu00003, 353398, Proteobacteria(100);Alphaproteobacteria(100);Caulobacterales(100);Caulobacteraceae(100);Brevundimonas(100);Brevundimonas_unclassified(100);Brevundimonas_unclassified(100);Brevundimonas_unclassified(100);

Otu00004, 159800, Firmicutes(100);Bacilli(100);Lactobacillales(100);Lactobacillaceae(100);Lactobacillus(100);Lactobacillus_unclassified(100);Lactobacillus_unclassified(100);Lactobacillus_unclassified(100);

Otu00005, 139955, Proteobacteria(100);Gammaproteobacteria(100);Enterobacteriales(100);Enterobacteriaceae(100);Enteric_Bacteria_cluster(100);Escherichia(96);Escherichia_unclassified(96);Escherichia_unclassified(96);

Otu00006, 114212, Firmicutes(100);Bacilli(100);Lactobacillales(100);Lactobacillaceae(100);Lactobacillus(100);Lactobacillus_unclassified(100);Lactobacillus_unclassified(100);Lactobacillus_unclassified(100);

Otu00007, 112891, Firmicutes(100);Clostridia(100);Clostridiales(100);Veillonellaceae(100);Veillonella(100);Veillonella_unclassified(100);Veillonella_unclassified(100);Veillonella_unclassified(100);

Otu00008, 109995, Firmicutes(100);Bacilli(100);Lactobacillales(100);Lactobacillaceae(100);Lactobacillus(100);Lactobacillus_unclassified(100);Lactobacillus_unclassified(100);Lactobacillus_unclassified(100);

Otu00009, 90539, Firmicutes(100);Bacilli(100);Lactobacillales(100);Lactobacillaceae(100);Lactobacillus(100);Lactobacillus_unclassified(100);Lactobacillus_unclassified(100);Lactobacillus_unclassified(100);

Otu00010, 86913, Cyanobacteria(100);Chloroplast(100);Chloroplast_unclassified(100);Chloroplast_unclassified(100);Chloroplast_unclassified(100);Chloroplast_unclassified(100);Chloroplast_unclassified(100);Chloroplast_unclassified(100);

Otu00011, 76139, Proteobacteria(100);Gammaproteobacteria(100);Enterobacteriales(100);Enterobacteriaceae(100);Enteric_Bacteria_cluster(100);Buttiauxella(94);Buttiauxella_unclassified(94);Buttiauxella_unclassified(94);

Otu00012, 60727, Firmicutes(100);Erysipelotrichi(100);Erysipelotrichales(100);Erysipelotrichaceae(100);Turicibacter(100);Turicibacter_unclassified(100);Turicibacter_unclassified(100);Turicibacter_unclassified(100);

Otu00013, 58728, Firmicutes(100);Bacilli(100);Lactobacillales(100);Lactobacillaceae(100);Lactobacillus(100);Lactobacillus_unclassified(100);Lactobacillus_unclassified(100);Lactobacillus_unclassified(100);

Otu00014, 49875,
Firmicutes(100);Bacilli(100);Lactobacillales(99);Enterococcaceae(92);Enterococcus(92);Enterococcus_unclassified(92);Enterococcus_unclassified(92);Enterococcus_unclassified(92);

Otu00015, 38064,
Firmicutes(100);Bacilli(100);Lactobacillales(100);Lactobacillaceae(100);Lactobacillus(100);Lactobacillus_unclassified(100);Lactobacillus_unclassified(100);Lactobacillus_unclassified(100);

Otu00016, 31302,
Actinobacteria(100);Actinobacteria(100);Actinobacteridae(100);Bifidobacteriales(100);Bifidobacteriaceae(100);Bifidobacteriaceae_unclassified(100);Bifidobacteriaceae_unclassified(100);Bifidobacteriaceae_unclassified(100);

Otu00017, 18817,
Actinobacteria(100);Actinobacteria(100);Actinobacteridae(100);Bifidobacteriales(100);Bifidobacteriaceae(100);Aeriscardovia(99);Aeriscardovia_unclassified(99);Aeriscardovia_unclassified(99);

Otu00018, 18267,
Actinobacteria(100);Actinobacteria(100);Actinobacteridae(100);Actinomycetales(100);Corynebacterineae(98);Corynebacteriaceae(92);Corynebacterium(92);Corynebacterium_unclassified(92);

Otu00019, 17251,
Actinobacteria(100);Actinobacteria(100);Actinobacteridae(100);Bifidobacteriales(100);Bifidobacteriaceae(100);Bifidobacterium(100);Bifidobacterium_unclassified(100);Bifidobacterium_unclassified(100);

Otu00020, 17019,
Synergistetes(100);Synergistia(100);Synergistales(100);Synergistaceae(100);Jonquetella(100);Jonquetella_unclassified(100);Jonquetella_unclassified(100);Jonquetella_unclassified(100);

Metabolic pathways 1-20 represented in Fig. 5.

PWY-7208	superpathway of pyrimidine nucleobases salvage
PWY-5667	CDP-diacylglycerol biosynthesis I
PWY0-1319	CDP-diacylglycerol biosynthesis II
PWY-6121	5-aminoimidazole ribonucleotide biosynthesis I
PWY-6122	5-aminoimidazole ribonucleotide biosynthesis II
PWY-6277	superpathway of 5-aminoimidazole ribonucleotide biosynthesis
PWY-7229	superpathway of adenosine nucleotides de novo biosynthesis I
PWY-7220	adenosine deoxyribonucleotides de novo biosynthesis II
PWY-7222	guanosine deoxyribonucleotides de novo biosynthesis II
PWY-7663	gondoate biosynthesis (anaerobic)
PHOSLIPSYN-PWY	superpathway of phospholipid biosynthesis III (E. coli)
NONOXIPENT-PWY	pentose phosphate pathway (non-oxidative branch) I
PWY-7219	adenosine ribonucleotides de novo biosynthesis
PWY-6126	superpathway of adenosine nucleotides de novo biosynthesis II
PWY-5686	UMP biosynthesis I
PWY4FS-7	phosphatidylglycerol biosynthesis I

PWY4FS-8	phosphatidylglycerol biosynthesis II
PWY-7221	guanosine ribonucleotides de novo biosynthesis
PWY-6387	UDP-N-acetylmuramoyl-pentapept biosynth I (meso-diaminopimelate contain.)
PWY-5973	cis-vaccenate biosynthesis
