# Supplementary figures and images for: The Impact of Physical Effort on the Gut Microbiota of Long-Distance Fliers
Source: Microorganisms. 2023 Jul 6;11(7):1766. doi: 10.3390/microorganisms11071766 (PMC10386721; doi:10.3390/microorganisms11071766)

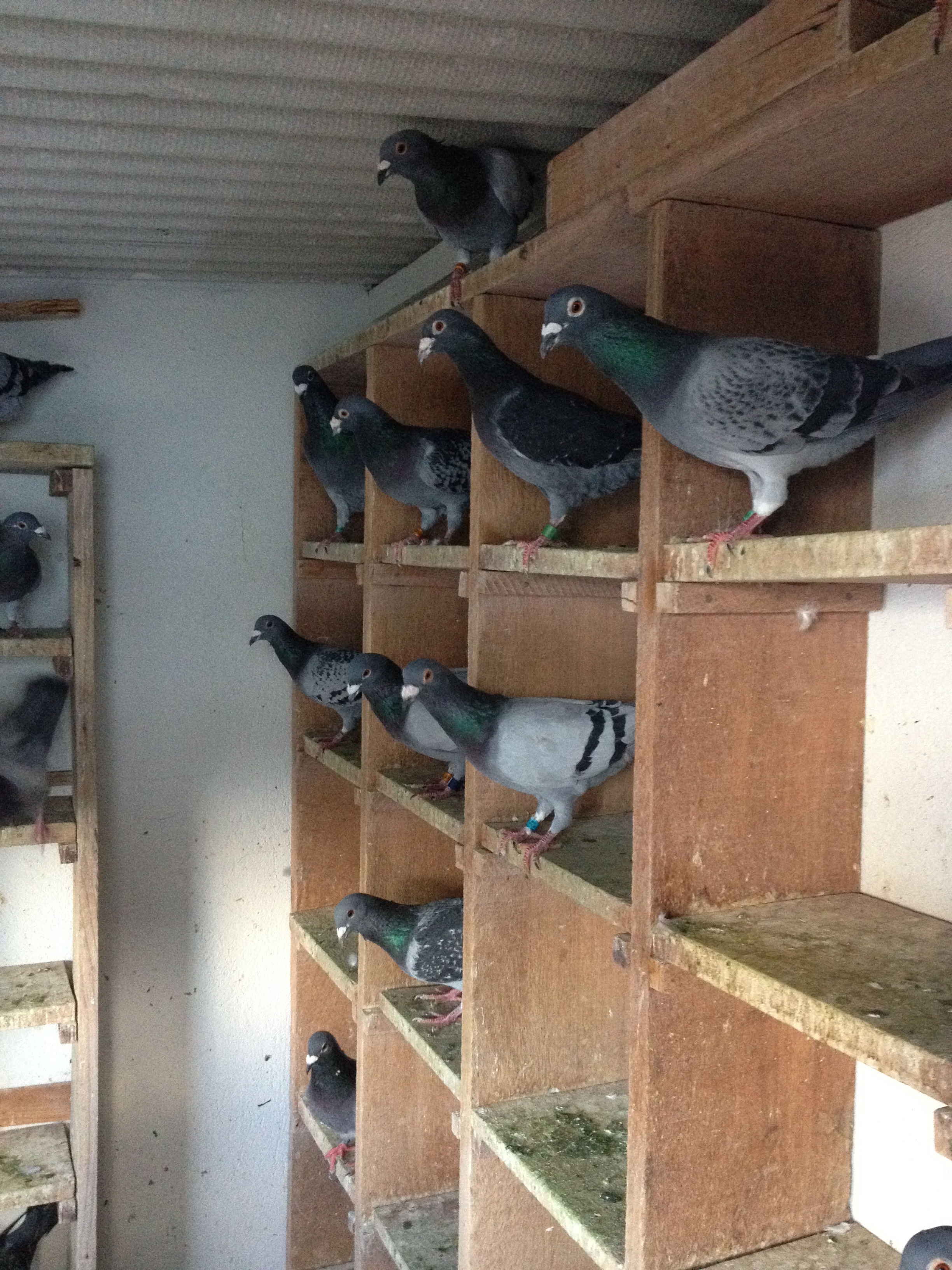

Supplement: Supplementary file 1 [file microorganisms-11-01766-s001.zip › FigureS1.jpg]
